# Development and disease-specific regulation of RNA splicing in cardiovascular system

**DOI:** 10.3389/fcell.2024.1423553

**Published:** 2024-07-09

**Authors:** Jinxiu Jiang, Hongchun Wu, Yabo Ji, Kunjun Han, Jun-Ming Tang, Shijun Hu, Wei Lei

**Affiliations:** ^1^ Department of Cardiovascular Surgery of the First Affiliated Hospital and Institute for Cardiovascular Science, State Key Laboratory of Radiation Medicine and Protection, Suzhou Medical College, Soochow University, Suzhou, China; ^2^ Hubei Key Laboratory of Embryonic Stem Cell Research, Hubei University of Medicine, Shiyan, China

**Keywords:** alternative splicing, cardiac development, cardiovascular disease, RNA binding protein, splicing factor

## Abstract

Alternative splicing is a complex gene regulatory process that distinguishes itself from canonical splicing by rearranging the introns and exons of an immature pre-mRNA transcript. This process plays a vital role in enhancing transcriptomic and proteomic diversity from the genome. Alternative splicing has emerged as a pivotal mechanism governing complex biological processes during both heart development and the development of cardiovascular diseases. Multiple alternative splicing factors are involved in a synergistic or antagonistic manner in the regulation of important genes in relevant physiological processes. Notably, circular RNAs have only recently garnered attention for their tissue-specific expression patterns and regulatory functions. This resurgence of interest has prompted a reevaluation of the topic. Here, we provide an overview of our current understanding of alternative splicing mechanisms and the regulatory roles of alternative splicing factors in cardiovascular development and pathological process of different cardiovascular diseases, including cardiomyopathy, myocardial infarction, heart failure and atherosclerosis.

## 1 Introduction

Cardiovascular diseases (CVDs) have been the leading cause of global mortality and morbidity for decades, and affect people of all ages (Marti-Gomez et al., 2022). Despite advancements in our understanding of gene expression patterns, our comprehension of the molecular mechanisms underlying the development of CVDs remains incomplete. Specifically, the role and regulation of various transcript isoforms generated by alternative splicing are poorly understood. Alternative splicing encompasses a variety of patterns, including mutually exclusive exons, exon inclusion/exclusion, and the retention of intronic sequences, resulting in the generation of multiple protein isoforms from a single gene (Park et al., 2018; Marasco and Kornblihtt, 2023).

Alternative splicing is pervasive across various organs, tissues, and cell types. Approximately 95% of human multiexon genes produce distinct transcripts through alternative splicing, thereby contributing to the diversity of genetic information transmission (Pan et al., 2008). Tissue-specific patterns of alternative splicing have been shown to coordinate the development of tissues and organs (Baralle and Giudice, 2017). Among them, cardiac development and heart diseases are particularly associated with alternative splicing. During cardiac development, RNA-binding proteins (RBPs) orchestrate the alternative splicing of sarcomere genes, so as to determine the structure and mechanical properties of cardiac muscle. Notably, Titin (TTN), the largest molecular spring in the heart encoded by 364 exons, is subjected to various alternative splicing events, thereby modulating the Titin-based passive tension, a determinant of diastolic function (Mehta and Touma, 2023). Splicing dysfunction has been suggested to be implicated in multiple diseases (Bonnal et al., 2020; Rogalska et al., 2023). This review focuses on recent advancements in understanding the role of alternative splicing in cardiac development and CVDs.

## 2 Alternative splicing and splicing factors

Transcriptional and post-transcriptional modifications play a crucial role in regulating gene expression in mammals. Among these modifications, RNA splicing is a highly regulated post-transcriptional modification process wherein introns are excised from the newly transcribed pre-mRNA, yielding mature mRNAs for protein synthesis. This intricate process, which primarily takes place in the nucleus, is broadly divided into two main types: constitutive splicing and alternative splicing. Constitutive splicing represents the default pathway, where all introns are uniformly removed from pre-mRNA, and exons are seamlessly joined together in the same order as they appear in the genome’s transcriptional sequence (Beqqali, 2018). In contrast, alternative splicing is a dynamic and finely controlled process that undergoes significant regulation during cellular differentiation or in response to specific physiological states. This dynamic regulation leads to the inclusion or exclusion of particular exons in unique combinations, resulting in the generation of multiple mRNA isoforms from a single gene (Zhu et al., 2017; Wright et al., 2022).

### 2.1 Types of alternative splicing

**FIGURE 1:**
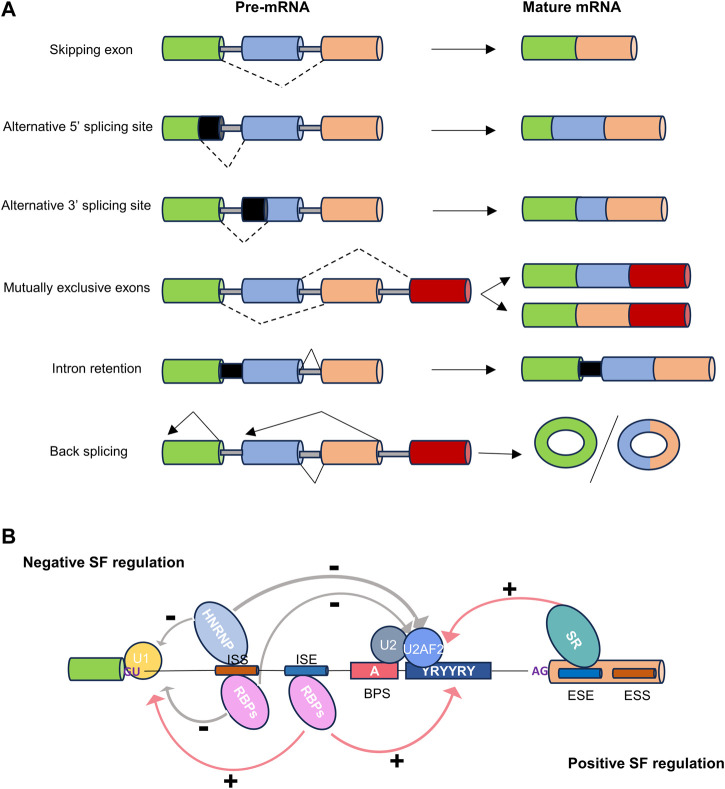
The types and regulation of alternative splicing. **(A)** Dot lines indicate alternative splicing. Alternatively spliced sequences can be classified into the following types: skipping exon, alternative 5′ or 3′splice site usage, inclusion of mutually exclusive exons, intron retention, and back splicing. **(B)** Cis-regulatory sequences necessary for splicing. The four basic splicing sequences are located in the 5′splice donor site (5′SS), the 3′splice acceptor site (3′SS), the branchpoint sequence (BPS), and the polypyrimidine tract (Py-tract). Regulatory splicing factors (SFs) acting as positive regulators (e.g., SR/other RBPs), or negative regulators (e.g., HNRNP/other RBPs), can bind to splice enhancer (ESE or ISE) or silencer (ESS or ISS) sequences in exons or introns to fine-tune splicing.

Alternative splicing can be categorized into five primary events based on the variation in splice site selection (Figure 1A). These variations in splice site selection can either shorten or lengthen exons, resulting in alternative 5′ splice site (A5′SS) and alternative 3′ splice site (A3′SS) splicing events. Splicing variation can also impact complete exons, which may be excluded from the mature transcript, referred to as skipping exon (SE) events. Mutually exclusive exons (ME) represent a complex scenario where one of two adjacent exons may be skipped, and both are rarely observed together in a mature transcript. Lastly, intron retention (IR) occurs when an intron is retained in a mature transcript. Different combinations of these primary events can lead to the production of multiple isoforms, resulting in changes to the untranslated regions (UTRs) or the coding sequence (Reixachs-Solé and Eyras, 2022). This, in turn, can elicit various functional consequences, including alternations in mRNA stability, localization and translation. Collectively, these modifications, coupled with changes in mRNA base sequences, could contribute to proteomic diversity or gene expression levels (Baralle and Giudice, 2017; Jacob and Smith, 2017).

In addition to the five most common primary events, there is a relatively new category of alternative splicing known as reverse splicing, which gives rise to circular RNAs (circRNAs) (Ule and Blencowe, 2019). Although Nigro et al. initially described circRNAs in 1991, they were largely overlooked due to their unconventional splicing behavior, where exons are joined at consensus splice sites but in a shuffled order compared to the primary transcript (Nigro et al., 1991). Until recent years, advances in sequencing technology have brought attention to circRNAs. It is believed that circRNAs are co-generated with mRNAs and play a role in regulating the expression of their pre-mRNA precursor, or regulating mRNA stability by competitively sequestrating miRNAs and derepressing their target mRNAs (Ashwal-Fluss et al., 2014).

### 2.2 Regulators of alternative splicing

The regulation of alternative splicing is orchestrated by multiple regulatory factors located at the exon-intron boundary, operating in either a cis or trans manner to precisely define splice sites (Figure 1B) (Gao and Wang, 2020). The two prominent families of RNA-binding proteins (RBPs) engaged in splicing regulation are serine- and arginine-rich proteins (SR) and heterogeneous nuclear ribonucleoproteins (hnRNPs). These two families typically play opposing regulatory roles in splicing. In addition to SR and hnRNP proteins, RBPs containing RNA recognition motifs (RRM), K homology structural domains (KH), and zinc finger structural domains also play a significant role in alternative splicing regulation (Mehta and Touma, 2023).

#### 2.2.1 SR family

The SR splicing factors (SRSF), also known as SR proteins, constitute a highly conserved family of RBPs that play crucial roles in the general splicing machinery. They are distinguished by their unique capability to interact simultaneously with RNA and other proteins. The canonical SR protein family comprises twelve members, denoted as SRSF1 through SRSF12. These proteins feature a common structural motif, typically encompassing an RNA recognition motif at the N-terminus, a glycine-rich spacer region, and a C-terminal domain rich in arginine and serine, often referred to as the RS domain (Wagner and Frye, 2021). The RS domain comprises at least 50 amino acids with over 40% RS dipeptide content and is inherently unstructured. This domain primarily facilitates protein-protein interactions with other proteins containing RS domains, although it has also been observed to interact with RNA. Notably, the reversible phosphorylation of numerous serine residues within the RS domain is of utmost importance for the regulation of SR protein activities. A fully phosphorylated RS domain is a prerequisite for the recruitment of SR proteins to transcription sites and spliceosome assembly, while RS dephosphorylation promotes splicing catalysis, mRNA packaging, and nuclear export (Wegener and Muller-McNicoll, 2019).

Typically, SR proteins bind to exon splicing enhancers and act as promoters of exon inclusion, with their effectiveness being influenced by their binding strength, expression levels, and interactions with other SR proteins and hnRNPs. However, it is noteworthy that SR proteins can occasionally exert repressive effects on splicing by binding to splicing silencer elements located near intron boundaries. Specifically, SRSF10, SRSF11, and SRSF12 emerge as general splicing repressors when they undergo dephosphorylation under specific stress conditions (Slišković et al., 2022).

#### 2.2.2 HNRNPs family

The hnRNP family comprises primarily 20 RNA-binding proteins, which are named alphabetically from hnRNPA1 to hnRNPU, as well as RALY that is also known as HNRPCL2 or P542. Four distinctive RNA-binding domains (RBDs), including the RNA recognition motif, the quasi-RRM, a glycine-rich domain constituting an RGG box, and a KH domain, were identified in hnRNP proteins (Wang et al., 2022).

The most prevalent RBD among hnRNP proteins is the RRM, found in more than 80% of all hnRNP proteins. The highly conserved octamer RNP1 and hexamer RNP2 sequences in RRM domains are crucial for their binding to RNA. Glycine-rich domain typically serves as an auxiliary domain, facilitating both homologous and heterologous interactions with other hnRNPs (Cartegni et al., 1996). Originally identified in hnRNPK, all KH domains within hnRNPs share a common structural motif comprising three-stranded antiparallel β-sheets packed against three α-helices (βααββα). A flexible loop connecting two α-helices in the KH core enables their interaction with RNA. In addition to above RNA-binding domains, hnRNPs frequently contain auxiliary domains, such as proline-rich, glycine-rich, or acid-rich domains. It is important to note that not all RBDs are exclusive, and the specificity of RNA binding is primarily mediated by the three-dimensional structure of the protein, where regions surrounding the RBDs finely tune the RNA-protein interactions (Geuens et al., 2016).

HnRNPs can bind to heterogeneous nuclear RNAs (hnRNAs) or pre-mRNAs, and are key regulators of alternative splicing (Wang et al., 2022). All members except hnRNP J/N/O/P/Q/R/S/T are involved in various aspects of alternative splicing (Geuens et al., 2016). Some hnRNPs bind to exon or intron splicing silencers, thereby often antagonizing the effect of SR proteins and inhibiting splicing. Similar to SR proteins, their regulatory role can shift depending on their relative position to other sequence elements, such as binding to intron splicing enhancers (Reixachs-Solé and Eyras, 2022).

#### 2.2.3 Other regulators

The RBFOX family of RNA-binding proteins, including RBFOX1, RBFOX2, and RBFOX3, plays a crucial role in the regulation of cell- or tissue-specific alternative splicing, miRNA processing, mRNA stability, and translation efficiency. These diverse functions of each RBFOX protein are facilitated by the spatiotemporal expression of distinct isoforms, either from different promoters or through alternative splicing mechanisms (Conboy, 2017). Each RBFOX protein features a single RNA recognition motif that enables its interaction with the (U)GCAUG element situated in alternatively spliced exons or in flanking introns. Typically, binding of RBFOX to the downstream of alternative exon promotes exon splicing, while binding to an upstream element or an element within the exon suppresses exon inclusion (Gu et al., 2020).

Beyond the RBFOX family, some RNA binding motif proteins (RBM) including RBM10, RBM20, RBM24, and RBM42 are involved in alternative splicing. RBM10, predominantly recognizing homopolymers of poly-uridine or -guanosine nucleotides, has been shown to regulate alternative splicing of apoptotic genes (Nanjo et al., 2022). Both RBM20 and RBM24 are responsible for the normal splicing of many cardiac genes and the formation of circRNAs (Khan et al., 2016; Fochi et al., 2020; Lu et al., 2022), while their deletion or mutation resulted in dilated cardiomyopathy (Guo et al., 2012; Nishiyama et al., 2022). Additionally, RBM42 can also modulate alternative splicing through interaction with hnRNPK, and play essential roles in neurological and myocardial functions (Chen et al., 2023).

The quaking (QKI) proteins, named QKI-5, 6 and 7, are derived from a single gene by alternative splicing, and are belonging to hnRNP K-homology (KH)-domain family. All QKI isoforms share an N-terminal KH domain flanked by QUA1 and QUA2 domains, but differ in their C-terminal ends. While cytoplasmic isoforms QKI-6 and QKI-7 regulate RNA stability and mRNA translation, the nuclear isoform QKI-5 is involved in alternative splicing of pre-mRNAs and mRNA nuclear export (Chen et al., 2021). Interestingly, the cytoplasmic QKI-6 can also regulate hnRNP F/H-dependent alternative splicing pathway in myelinating glia (Mandler et al., 2014).

Muscle blind-like proteins (MBNL) are alternative splicing factors implicated in myotonic dystrophy. MBNL proteins are encoded by the MBNL1, MBNL2, and MBNL3 genes in mammals, and share four highly conserved zinc-finger domains (ZnF), which are responsible for recognizing specific pre-mRNA and mRNA targets. These proteins exhibit dual functions, serving as either repressors or activators of splicing in various transcripts (Gonzalez et al., 2022).

In addition to the aforementioned splicing factors, certain transcription factors have also been implicated in regulating alternative splicing in cardiac development (Han et al., 2017). A recent study by Zhu et al. demonstrated that GATA4, a zinc-finger containing DNA binding transcription factor essential for normal cardiac development and homeostasis, can regulate cell type-specific alternative splicing. This regulation occurs through interactions with members of the spliceosome complex and sequence-specific interactions with a large number of mRNAs (Zhu et al., 2022). Knockdown of GATA4 in human cardiac progenitors resulted in 1,599 differential alternative splicing events occurring over 1,247 genes. This novel paradigm has deepened our understanding of tissue-specific regulatory mechanisms of RNA splicing. However, many more tissue-enriched transcriptional factors regulating RNA splicing remain to be characterized.

#### 2.2.4 Circular RNAs

Circular RNAs (circRNAs) are covalently closed noncoding RNAs generated via back-splicing of pre-mRNAs, an additional type of alternative splicing. The biogenesis of circRNAs is catalyzed by canonical spliceosome machinery, and modulated by both intronic complementary sequences and RNA binding proteins such as QKI, hnRNPL and RBM20.

In addition to serving as miRNA sponge, a growing body of evidence underscores the pivotal role of circRNAs as molecular regulators of RNA transcription and splicing (Li et al., 2018). For instance, CircSEP3, a nuclear retained circRNA derived from exon 6 of SEPALLATA3 (SEP3) in Arabidopsis, has been shown to bind strongly to its cognate DNA locus, resulting in transcriptional pausing of the linear RNA and formation of alternatively spliced SEP3 mRNA with exon skipping (Conn et al., 2017). Meanwhile, Muscle blind (MBL) gene-derived circMBL was reported to exert an autoregulatory influence on the alternative splicing of MBL mRNA, subsequently governing physiological processes (Ashwal-Fluss et al., 2014; Pamudurti et al., 2022). Above evidences suggest that circRNAs modulate gene expression at both transcription and post-transcription levels.

## 3 Alternative splicing in cardiovascular development

### 3.1 Alternative splicing in cardiac development

The coordinated control of alternative splicing plays a pivotal role in numerous developmental processes by modifying the transcriptome and, consequently, the proteome. Extensive evidence from animal model studies underscores the significance of proper splicing in facilitating optimal heart development (Nagasawa et al., 2018). Alternative splicing orchestrates the expression of protein isoforms, thereby enabling the adaptation of cardiac function during both development and disease. A multitude of essential genes involving in heart development are subject to regulation through alternative splicing. These genes include those encoding structural sarcomere proteins such as TTN, TnT, Myom1 (myomesin-1), TPM1, among others. Additionally, genes related to ion channels critical for myocardial contraction, including CACNA1C, CAMK2D, RYR2, SCN5, ATP2A2, as well as other vital genes associated with cardiac development, are subject to alternative splicing regulation (Table 1).

**TABLE 1:** Splicing factors and their targets in heart development and diseases.

Splicing factor	Main Target(s)	Role in heart development and diseases
RBM20	TTN, Ca_V_1.2, CamkIIδ, RYR2, CACNA1C	Sarcomere assembly, Ca^2+^ regulation, excitation-contraction, and rescue of the cardiac dysfunction in mice (George et al., 2007; Guo et al., 2012; Khan et al., 2016; Duran et al., 2021)
RBM24	Tpm2, TTN, Nebl, Fhod3, Enah, Ablim1	Sarcomere structure (Liu et al., 2019)
RBFOX1	Ca_V_1.2, CamkIIδ, Ketohexokinase, MEF2	Smooth muscle and myocardial contraction, Ca^2+^ regulation. Deficiency-induced isoform switch of the MEF2 contributes to CVDs (Guo et al., 2012; Gao et al., 2016; Duran et al., 2021)
RBFOX2	TPM1, Ca_V_1.2, CamkIIδ	Myofibril organization, myocardial contraction, and Ca^2+^ regulation (Guo et al., 2012; Cao et al., 2021; Duran et al., 2021)
SRSF1	CamkIIδ, VEGF-A	Ca^2+^ regulation, angiogenesis (Guyot and Pagès, 2015; Duran et al., 2021)
SRSF2	RYR2, VEGFR1	Excitation–contraction, angiogenesis (George et al., 2007; Abou Faycal et al., 2019)
SRSF5	Mmp9, Mmp12, Fgf16, VEGF-A	Cell proliferation, angiogenesis (Guyot and Pagès, 2015)
SRSF6	cTnT, FGFR, VEGF	Calcium regulation of actin filament function, myocardial contraction, angiogenesis and myocardial infarction (Jin and Cote, 2004; Guyot and Pagès, 2015; Lu and Yin, 2016; Jiao et al., 2022)
hnRNPD	VEGFR1	Angiogenesis (Ikeda et al., 2011)
hnRNPU	Junctin	Affect calcium dynamics in cardiomyocytes (Ye et al., 2015)
MBNL	cTnT, SCN5A	Myocardial contraction, cardiac conduction (Freyermuth et al., 2016; Marques and de Oliveira, 2016; Lopez-Martinez et al., 2020)
PTBP1	FGFRs	Angiogenesis. Overexpression of PTBP1 induces cardiac hypertrophy and diastolic dysfunction (Bruno et al., 2004; Marti-Gomez et al., 2022)
QKI5	circRNAs	Prevented expansion of cardiomyocytes in cardiomyopathy (Gupta et al., 2018)
SF3B1	Ketohexokinase	Pathological cardiac hypertrophy (Mirtschink et al., 2015)
SLM2	TTN	Deficiency of SLM2 leads to heart failure (Boeckel et al., 2022)

#### 3.1.1 Sarcomere genes

##### 3.1.1.1 Titin

Titin protein, encoded by TTN gene, is a critical component essential for myofibril elasticity and the structural integrity of the sarcomere (Lopez-Martinez et al., 2020). The TTN pre-mRNA undergoes a multitude of alternative splicing events, giving rise to several tissue-specific and developmentally regulated isoforms of titin. Among the well-characterized isoforms are those specific to cardiac and skeletal muscle, named N2A, N2B, and N2BA. During cardiac development, there is a gradual increase in the frequency of TTN exon skipping, favoring the production of shorter isoforms (Opitz and Linke, 2005). This shift in protein expression, transitioning from the long elastic TTN isoform N2A to the shorter, stiffer isoforms N2B and N2BA, is precisely coordinated through alternative splicing (Fochi et al., 2020). A key regulator in this process is RBM20, which primarily governs heart-specific TTN pre-mRNA splicing, and modulate the ratio of TTN isoforms N2BA to N2B during both cardiac development and adult cardiac function. Recently, Khan et al. revealed an essential role of RBM20 in the production of circRNAs from the TTN I-bind, a region subjected to extensive regulation through alternative splicing (Khan et al., 2016). However, splicing of TTN is a complex process. Aside from RBM20, TTN shifts towards the N2BA isoform due to inclusion of several immunoglobin domains, the N2A and the PEVK regions were observed in the QKI knockout mice (Chen et al., 2021; Montanes-Agudo et al., 2023). Additionally, investigations in knockout mice also indicated the involvement of MBNL, RBM2 and RBFOX2 in splicing, by regulating exon skipping or inclusion (Lopez-Martinez et al., 2020; Montanes-Agudo et al., 2023).

##### 3.1.1.2 Cardiac troponin T

Cardiac troponin T (cTnT) is the anchoring subunit of troponin complex situated on the thin filament, and is a pivotal component responsible for the calcium regulation of actin filament function and cardiac muscle contraction. Four isoforms of cTnT are expressed in the human heart due to alternative splicing of exons 4 and 5 of the TNNT2 gene (Gao and Wang, 2020). Interestingly, exon 5 encoding 10 amino acids in the NH_2_-terminal region is included only in the fetal and neonatal cTnT isoforms but excluded from the adult cTnT isoforms. Meanwhile, the structural variations in the NH_2_-terminal region of cTnT result in significant changes in the sensitivity of cTnT to Ca^2+^ and activation of the actomyosin ATPase (Sheng and Jin, 2014). In contrast, alternate splicing of exon 4 appears to be independent of developmental stages, and results in comparatively smaller structural and functional variations. The splicing regulator SRSF6 is responsible for the inclusion of exon 5 into cTnT, whereas Dyrk1A (dual-specificity tyrosine phosphorylation-regulated kinase 1A) promotes exon 5 inclusion by phosphorylating the SRSF6 protein (Lu and Yin, 2016). Contrary to SRSF6 protein, MBNL was reported to promote the exclusion of exon 5 during heart development (Kalsotra et al., 2008). Taken together, these observations underscore the significance of alternative splicing-mediated structural changes in the cTnT protein for its functional adaptation during heart development.

##### 3.1.1.3 Tropomyosin 1

Tropomyosin 1 (TPM1) is the predominant tropomyosin gene expressed in cardiac muscle, playing a significant role in muscle contraction (Bai et al., 2013). Its presence is essential for myofibril organization, myocardial contraction, and proper cardiac development. Mutations or abnormal expression of TPM1 have been linked to familial hypertrophic cardiomyopathy, dilated cardiomyopathy, and heart failure (Karam et al., 2011; Marques and de Oliveira, 2016). TPM1 comprises 15 exons in rats, several of which undergo alternative splicing transitions during embryonic heart development, leading to the predominant generation of muscle-specific TPM1 isoforms in adult hearts. Although both RBFOX2 and the polypyrimidine tract-binding protein 1 (PTBP1) are highly expressed in embryonic hearts, they oppositely regulate the alternative splicing of rat Tpm1 exon 6a, highlighting the strict regulation of TPM1 splicing isoforms during rat cardiac development (Cao et al., 2021).

#### 3.1.2 Excitation–contraction coupling-related genes

##### 3.1.2.1 L-type calcium channel

L-type Ca_V_1.2 channels are high voltage-activated channels encoded by the CACNA1C gene, and serve as the primary conduits for Ca^2+^ influx to initiate smooth muscle and myocardial contraction. In humans, at least 20 of 55 exons undergo alternative splicing, raising more than 50 Ca_V_1.2 splice combinations found in the heart and smooth muscles. These splicing events have a profound impact on the function CACNA1C by altering electrophysiology properties, Ca^2+^ dependency, channel regulation, affinity of dihydropyridines and even leading to the loss of channel functions (Liao et al., 2015). Importantly, these functionally diverse Ca_V_1.2 subtypes are developmentally regulated in heart, while some neonatal splice variants of the Ca_V_1.2 channels are re-emerged in chronic heart diseases. While splice factors, including RBFOX1, RBFOX2 and PTBP1, have been shown to regulate Ca_V_1.2 splicing in mouse cortex, deep sequencing of cardiac transcriptome validated RBM20-dependent regulation of Ca_V_1.2 splicing at exon 8, 9, 22 and 31 in the heart or during human cardiogenesis from pluripotent stem cells (Guo et al., 2012; Hu et al., 2017; Bertero et al., 2019). However, more experiments are necessary to investigate the cardiac-specific CaV1.2 splicing regulated by other splicing factors.

##### 3.1.2.2 Calcium/calmodulin dependent kinase

Ca^2+^/calmodulin dependent protein kinase II delta (CAMKIIδ) belongs to the multifunctional CaMK family, and is best known to participate in regulation of Ca^2+^ handling in cardiomyocytes. CAMK2D is the gene responsible for encoding the heart ion channel-related protein CamkIIδ. During cardiac development, CamkIIδ exhibits expression in multiple subtypes, and changes in the expression of CAMK2D variant have been linked to cardiac hypertrophy and heart failure. The known splicing factors targeting CamkIIδ include RNA-binding proteins RBFOX1, RBFOX2, SRSF1 and RBM20 (Duran et al., 2021). Cardiomyocytes from either Srsf1^−/−^ or Rbm20^−/−^ mice display aberrant splicing events, resulting a shift from the smaller isoforms CaMKIIδB/δC to the largest isoform CaMKII-δA, characterized by the inclusion of exon 15 and 16 but not exon 14. This alteration affects their relative intracellular distributions and leads to Ca^2+^ handling disturbances (van den Hoogenhof et al., 2018). While cardiac specific knockout of Rbfox1 or Rbfox2 induces CaMKIIδ mis-splicing, RBM20 knockout leads to increased expression of CaMKIIδA and CaMKIIδ9, resulting in Ca^2+^ handling disturbances as shown in SRSF1 knockout cardiomyocytes.

##### 3.1.2.3 Cardiac ryanodine receptor and SR-Ca^2+^ ATPase

In cardiac muscle, the cardiac ryanodine receptor (RYR2) and sarco/endoplasmic reticulum (SER) Ca^2+^-ATPase type 2 (SERCA2) are pivotal components in sarcoplasmic reticulum-mediated Ca^2+^ cycling. They play crucial roles in the coordination of excitation-contraction coupling, with RYR2 facilitating Ca^2+^ release and SERCA2 responsible for Ca^2+^ uptake. Two splicing variants of RYR2 carrying 24 bp or 30 bp insertions, regulated particularly by RBM20 and SRSF2, show distinct cellular localization and modulation of Ca^2+^ release (George et al., 2007). Transcripts originating from the SERCA2 gene also undergo alternative splicing in a developmentally regulated and tissue-specific manner. This splicing process results in the formation of two isoforms: SERCA2a, predominantly expressed in slow-twitch (type 1) skeletal and cardiac muscles, and SERCA2b, which exhibits a broader distribution across tissues. The switch from SERCA2a to SERCA2b in mice hearts leads to reduced calcium uptake, compromised contractility and relaxation, and developmental cardiac defects (Ver et al., 2001).

##### 3.1.2.4 Sodium channel protein type 5 subunit alpha

SCN5A encodes the α-subunit of the cardiac voltage channel NaV1.5, a crucial component responsible for the excitability of cardiomyocytes and rapid propagation of the impulse through the cardiac-conduction system. The regular developmental transition of SCN5A is regulated by MBNL1, resulting in replacement of exon 6A in fetal heart by exon 6B in the adult heart. Mice with exon 6B deletion exhibited a shift in SCN5A splicing, leading to the inclusion of the fetal exon 6A and the development of cardiac conduction defects (Freyermuth et al., 2016; Pang et al., 2018).

#### 3.1.3 Other heart development-related genes

In addition to the above-mentioned sarcomere structural proteins and ion channel-related genes, there are many genes involved in heart development are subjected to splicing regulation to fulfill their specific functions. SRSF5 has been reported to influence the mRNA levels of such as Mmp9, Mmp12, and Fgf16, which play pivotal roles in cell proliferation (Dwivedi et al., 2009). SRSF10 also plays a role in the muscle-specific splicing of Lrrfip1 pre-mRNA by promoting the inclusion of exons 16 and 17, an essential event in mouse heart development and myoblast differentiation (Wei et al., 2015). Moreover, SRSF10 controls the alternative splicing of genes such as Fxr1, Mef2a, and Nasp, expressed in heart ventricles. Recent evidence indicates a back splicing event occurring on the titin pre-mRNA, giving rise to a circular RNA (cTTN1) harboring an SRSF10 binding site, whereas loss of cTTN1 leads to abnormal splicing of SRSF10 targets like MEF2A and CASQ2 (Tijsen et al., 2021). Furthermore, Liu et al. demonstrated that RBM24 deletion in mice resulted in the mis-splicing of several genes coding for sarcomere structure proteins, including Tpm2, TTN, Nebl, Fhod3, Enah and Ablim1 (Liu et al., 2019). These evidences indicate the occurrence of extensive RNA variable splicing events during cardiac development.

### 3.2 Alternative splicing in angiogenesis

Angiogenesis, defined as the formation of new blood vessels from the existing vascular system, is an essential biological process for a proper development of a functional circulatory system providing oxygen and nutrients to every cell of the body. Numerous signaling molecules involved in the angiogenesis undergo alternative splicing events during embryonic development, among which include vascular endothelial growth factor-a (VEGF-A) and receptors, fibroblast growth factor (FGF), angiopoietin-2 (ANG-2), resulting in the generation of tissue-specific and development-stage-specific expression of distinct variants (Bowler and Oltean, 2019).

#### 3.2.1 Vascular endothelial growth factor A and receptors

*In vivo*, VEGF-A serves as the principal stimulatory signal for angiogenesis by binding to its receptors (VEGFR1 and VEGFR2) in endothelial cells. The splicing of VEGF-A pre-mRNA generates a variety of isoforms, whereas VEGF-A_111_, VEGF-A_121_, VEGF_145_, VEGF-A_165_, VEGF-A_189_ and VEGF-A_206_ are the six major isoforms, affecting endothelial cell proliferation, adhesion, and migration. Among them, VEGF-A_165_ is both the most abundantly expressed and the potent initiator of angiogenesis. Specifically, another family of VEGF-A isoforms was discovered nearly two decades ago. These isoforms, including VEGF-A_165_b, VEGF-A_121_b, VEGF-A_189_b, and VEGF-A_145_b, result from the utilization of a distal 3′splice site in exon 8, distinct from the isoforms mentioned earlier (VEGF-A_xxx_a), which are spliced at the proximal splice sites. These VEGF-A_xxx_b isoforms display an anti-angiogenic property, and have not been detected in endothelial cells (Stevens and Oltean, 2019). Currently, SR proteins, including SRSF1, SRSF2, SRSF5 and SRSF6, have been reported to play a role in the alternative splicing of VEGF-A pre-mRNA (Guyot and Pagès, 2015). The nuclear localization of SRSF1 after serine-arginine protein kinase 1-mediated phosphorylation promotes the production of the pro-angiogenic isoform VEGF-A_165_. Moreover, both SRSF2 and SRSF6 favor the generation of VEGF-A_xxx_b isoforms, whereas knockdown of SRSF1 altered the splicing of VEGF-A mRNA to produce more of the VEGF-A_xxx_b isoforms.

When compared to VEGFR1, VEGFR2 has significantly enhanced kinase activity, even though it has a relatively lower affinity for the binding of VEGF-A. Consequently, VEGFR2 is widely regarded as the primary functional receptor, while VEGFR1 is regarded as a decoy receptor. The splicing of VEGFR2 further complicates the VEGF signaling pathway. The soluble isoform of VEGFR2 (sVEGFR2), arising from partial retention of intron 13 and a consequential early termination stop codon, has a unique c-terminal sequence that does not exist in the membrane-bound VEGFR2 (Pavlakovic et al., 2010). This sVEGFR2 can prevent lymphangiogenesis through trapping VEGF-C and inhibit VEGF-C-mediated lymphatic endothelial cell proliferation.

Similar to VEGFR2, VEGFR1 exist as either membrane-bound or soluble molecules, which are controlled by alternative splicing. Four soluble isoforms of VEGFR1, including sVEGFR1-i13, sVEGFR1-i14, sVEGFR1-e15a and sVEGFR1-e15b, have been discovered shown to play an anti-angiogenic role. Numerous studies have provided evidences for a role of hnRNPD and SRSF2 in splicing regulation of VEGFR1. HnRNPD significantly decreases the sVEGFR1/mVEGFR1 (membrane-bound VEGFR1) ratio in HUVECs (Ikeda et al., 2011), but promotes the sVEGFR1 expression in human macrophage-like U937 cells (Fellows et al., 2013), indicating cell type-specific effects of hnRNPD on VEGFR1 splicing. Meanwhile, SRSF2 was recently shown to promote the splicing of VEGFR1 to the sVEGFR1-i13 isoform (Abou Faycal et al., 2019).

#### 3.2.2 Fibroblast growth factor and receptors

Fibroblast growth factor (FGF) family members, particularly FGF-1 and FGF-2, play an important role in angiogenesis, through binding to four receptor tyrosine kinases (FGFR1-4) and heparan sulfate proteoglycans (HSPGs). Alternative splicing of the last portion of the immunoglobulin-like domain 3 (IgIII) in the c-termini of FGFR1-3 is the most described event in the literature. Many RBPs are involved in the regulation of IgIII c-terminal splicing by mutually exclusive usage of either exon 8 or exon 9, giving rise to IIIb and IIIc isoforms (Di Matteo et al., 2020). While both ESRP1 and ESRP2 promote the expression of the IIIb isoform, hnRNPA1 and PTB are associated with the silencing of IIIb. Additionally, hnRNP F/H/K and forkhead box 2 (FOX-2) repress the expression of the IIIc isoform. FGFR1IIIc, FGFR2IIIc, and FGFR3IIIc are the primary isoforms expressed in endothelial cells, whereas an unbalance of FGFR-III splicing isoforms is involved in tumor angiogenesis (Hovhannisyan and Carstens, 2007; Liang et al., 2015).

The inclusion or exclusion of exons encoding the IgI and acid box domains is also a primary splicing event for FGFRs. The excision of the IgI domain give rise to FGFRβ, whereas the inclusion of the exon produces FGFRα. Recently, PTBP1 splicing repressor is involved in regulating the alternative splicing of FGFR1α and FGFR1β. It has been reported that PTBP1 induces α exon deletion by binding to intron splicing silencer sequences flanking α exons, leading to positive regulation of FGFR1β (Bruno et al., 2004). Therefore, further studies are needed to elucidate the exact role of PTBP1 in regulating FGFR1α/FGFR1β splicing. Furthermore, knockdown of SRSF6 leads to increased expression of FGFR1α (Jin and Cote, 2004).

#### 3.2.3 Hypoxia-inducible factor-α

Hypoxia-inducible factor-α (HIF-α) is responsible for various responses to hypoxia, including the promotion of angiogenesis to provide oxygen to cells in low-oxygen conditions. When forming a dimer with HIF-β, HIF-α binds to HIF response cis-elements, thereby enhancing the transcription of target genes, including VEGF-A. Three homologs of HIF-α and HIF-β have been identified and designated HIF-1α and HIF-1β, HIF-2α and HIF-2β, and HIF-3α and HIF-3β.

HIF-1α Δ11, HIF-1α Δ12, HIF-1α Δ11&12, HIF-1α Δ14, and HIF-1α^417^ are splice isoforms of HIF-1α arising from cassette exon skipping (Bowler and Oltean, 2019). Due to lack of domains required for transcription of HIF-target genes, most of these isoforms are predicted to play an inhibitory effect on HIF signaling in normoxia condition. As a major negative regulator of HIF-1α, HIF-3α is has also been reported to undergo alternative splicing (Cha et al., 2009). The hypoxia-inducible inhibitory Per/Arnt/Sim (IPAS) is the most defined alternative splicing isoform of HIF-3α. In the corneal epithelium, IPAS has been found to negatively regulate VEGF-A gene expression, indirectly inhibiting angiogenesis. Another splice variant of HIF-3α, known as HIF-3α4, is produced by the inclusion of intron 7, and has been demonstrated to impede angiogenesis and proliferation (Ando et al., 2013).

#### 3.2.4 Angiopoietin

Angiopoietins including Ang-1, Ang-2, and Ang-3/4 are involved in endothelial cell survival and vascular maturation. Smooth muscle cell or perivascular cell-derived Ang-1 can interact with endothelial cell-specific tyrosine kinase (Tie-2) receptor, resulting in phosphorylation and activation of Tie-2, and subsequently enhancing the stabilization of new blood vessels. On the other hand, Ang-2 is primarily released from endothelial cells and acts as a pro-angiogenic factor by antagonist against the activation of Tie-2 by Ang-1 (Thomas and Augustin, 2009).

The alternative splicing of Ang-1 and Ang-2 gives rise to both pro- and anti-tumorigenic isoforms. Exon 7 in Ang-1 is responsible for receptor binding, whereas the shorter versions lacking this domain may act as inhibitors of Ang-1 signaling. The Ang-2B variant, arising from the inclusion of exon 1B instead of exon 1, is first discovered in chicken and showed to be more highly expressed in adult quiescent testis than the canonical isoform, which is abundant in immature testis and fully regressed testis. It is considered that Ang-2B may be involved in inactivating the vasculature in quiescent testis (Mezquita et al., 1999). Ang-2_443_, also known as Ang-2C, is another splice variant of Ang-2, arising from the skipping of exon 2. This isoform can bind to but not activate Tie-2, and thus act as a negative regulator of Tie-2 signaling in during tumorigenesis and inflammation (Kim et al., 2000).

### 3.3 CircRNAs in cardiovascular development

As a relatively new category of alternative splicing, circRNAs have been increasingly identified through RNA sequencing data during cardiovascular development, particularly at various stages of cardiovascular lineage cell differentiation (Lei et al., 2018). Compared to pluripotent stem cells, differentiated cardiomyocytes exhibit a significantly higher abundance of circRNAs, such as circSLC8A1-1, circTTN-275, and circALPK2-1. These circRNAs show strong positive correlations with the time course of cardiomyocyte differentiation, suggesting that their crucial roles in cardiac cell differentiation and function (Devaux et al., 2015; Tan et al., 2017; Lei et al., 2018).

Recent study revealed that the super-enhancer-regulated circNfix inhibits cardiomyocyte proliferation by inducing the ubiquitin-dependent degradation of Ybx1, ultimately repressing the expression of cyclin A2 and cyclin B1 (Huang et al., 2019). In the context of angiogenesis, Garikipati et al. found that circFndc3b binds to FUS (fused RNA-binding protein in sarcoma), a regulator of VEGF, to modulate VEGF expression and signaling. Overexpression of circFndc3b in cardiovascular endothelial cells increases VEGF-A expression, enhances its angiogenic activity, and reduces endothelial cell apoptosis (Garikipati et al., 2019).

These findings highlight the physiological role of circRNAs in cardiac development and neovascularization. Despite these advancements, the investigation of the functions and mechanisms of circRNAs in cardiovascular development are still in its infancy. Further functional studies are essential to elucidate the precise mechanisms by which circRNAs function in cardiac development and function.

## 4 Alternative splicing in cardiovascular diseases

**FIGURE 2:**
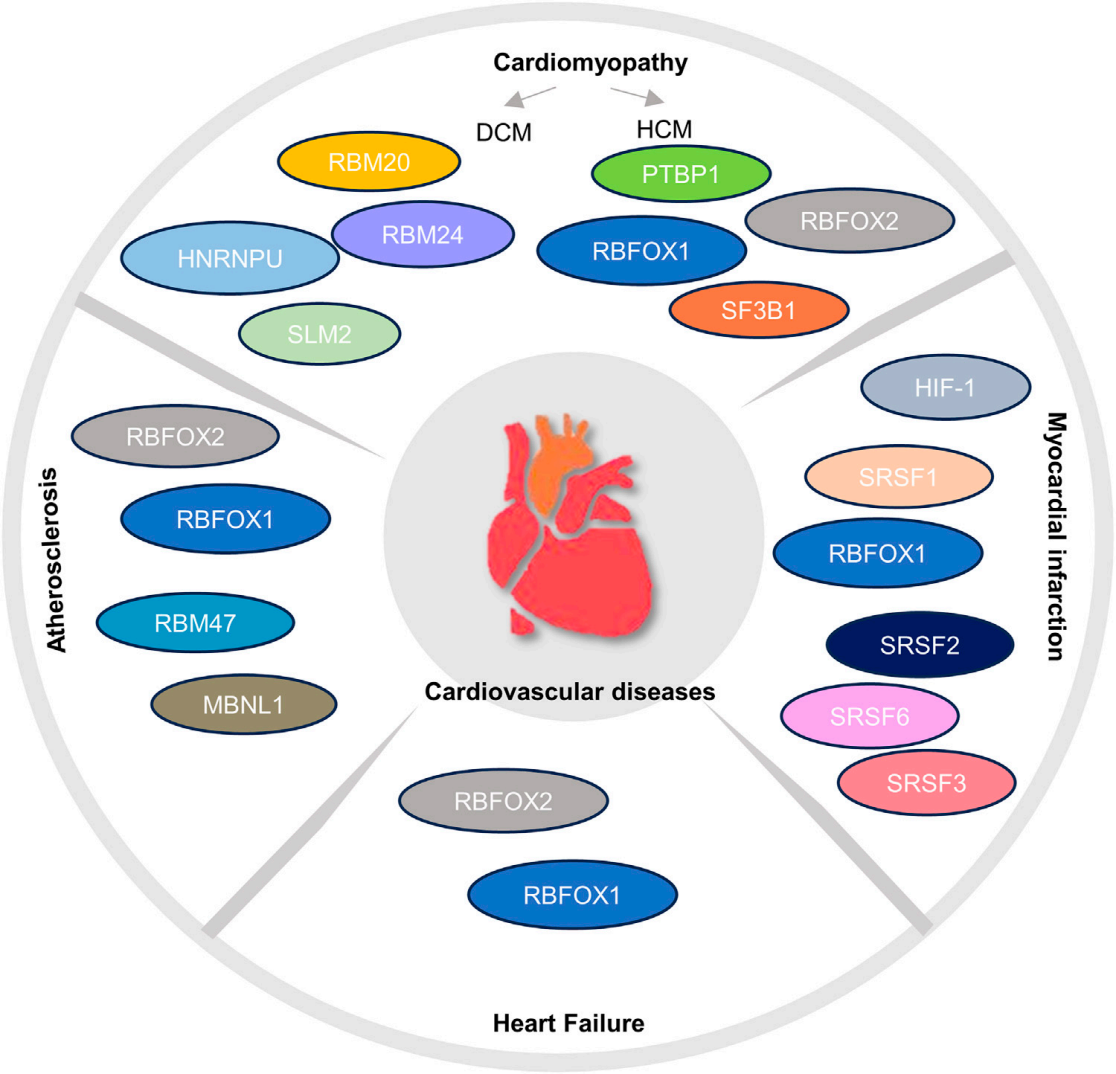
Regulatory factors of alternative splicing in cardiovascular diseases.

In recent years, splicing analysis has become a crucial focus in cardiovascular risk research. Numerous studies have provided compelling evidence that the regulation of splicing and alternative splicing events plays a pathogenic role in cardiac development and cardiovascular disease. This is primarily attributed to the fact that the dysregulation of cardiac splicing factors has a detrimental impact on cardiac function and contributes to the onset and progression of diseases in the cardiovascular system (Bryzgalov et al., 2022; Gotthardt et al., 2023). Notably, circRNAs have also been implicated in cardiac pathology. A study utilizing ribosomal deletion RNA sequencing identified six circRNAs (cTTN1, cTTN2, cTTN3, cTTN4, cTTN5, and cCAMK2D) that were significantly downregulated in patients with dilated cardiomyopathy, and one circRNA (cCAMK2D) that was downregulated in patients with hypertrophic cardiomyopathy (Khan et al., 2016). The following section focuses on the regulatory role of alternative splicing in four cardiovascular diseases: cardiomyopathy, myocardial infarction, heart failure, and atherosclerosis (Figure 2; Table 1).

### 4.1 Cardiomyopathy

Cardiomyopathy refers to a myocardial disease characterized by structurally and functionally abnormal, in the absence of coronary artery disease, hypertension, valvular disease and congenital heart disease sufficient to cause the observed myocardial abnormality (McKenna et al., 2017). The most prevalent forms of cardiomyopathies are hypertrophic cardiomyopathy (HCM), characterized by the thickening (hypertrophy) of the heart muscle, and dilated cardiomyopathy (DCM), marked by the stretching and thinning (dilation) of the heart muscle. Both conditions make it challenging for the heart to effectively pump blood (Watanabe et al., 2018).

#### 4.1.1 Hypertrophic cardiomyopathy

As the most common inherited heart disease, HCM is predominantly caused by mutations in genes. More than half of the HCM patients carry nonsense, frameshift, or missense mutations in one of eight sarcomere genes, while mutations in MYH7 and MYPBC3 occur the most often (Sabater-Molina et al., 2018). Advancements in RNA analysis have revealed that mutation-induced defects in RNA splicing are a significant contributor to HCM. A majority of splice site variants associated with HCM are located in the two most highly conserved nucleotide positions at the splice junction of MYPBC3 (Ribeiro et al., 2020). Moreover, multiple variants located in the near-splice-site region are shown to either activate cryptic splice site or induce exon skipping. However, whether these alternative splicing events mediate the mutation-induced HCM is still being explored.

Some RNA splicing regulators have been involved in the development of HCM. Ketohexokinase, the central fructose-metabolizing enzyme in heart, is shown to be alternatively spliced during pathological cardiac hypertrophy by the splicing factor 3b subunit 1 (SF3B1) (Mirtschink et al., 2015). RBFOX1 deficiency-induced isoform switch of the transcription factor MEF2 contributed to cardiomyocyte hypertrophy and pathological gene in the pressure overload mouse model (Gao et al., 2016). Moreover, recent studies have demonstrated that the overexpression of PTBP1 was sufficient to induce cardiac hypertrophy and diastolic dysfunction. These effects are believed to be partially mediated by the induction of a limited number of splicing changes (Marti-Gomez et al., 2022).

As a product of alternative splicing, HRCR is the first circRNA identified to play a functional role in cardiac hypertrophy. The expression of HRCR was in downregulated in both isoproterenol or transverse aortic constriction-induced mouse models of hypertrophy. Through interacting with miR-233*, HRCR was found to regulate the expression level of apoptosis repressor with CARD domain (ARC), which mediates cellular hypertrophy (Wang et al., 2016). Additionally, circSLCA8A1 has been suggested as a potential therapeutic target for cardiac hypertrophy. CircSLC8A1 is a circRNA highly expressed in cardiomyocytes and is abnormally upregulated in patients with DCM (Lim et al., 2019). These findings highlight the significance of circRNAs in cardiac pathology.

#### 4.1.2 Dilated cardiomyopathy

DCM is characterized by the dilation of the left ventricle of the heart and systolic dysfunction. It is a highly prevalent disease that can eventually lead to heart failure or sudden cardiac death. About half of DCM cases have a positive family history with an autosomal pattern of inheritance. Genes having definitive evidence of mutations associated with DCM include TTN, LMNA, MYH7, FLNC, BAG3, TNNT2, RBM20, SCN5A, DES, PLN and TNNC1 (Micolonghi et al., 2023).

Mutations in the splicing factor RBM20 are responsible for approximately 2%–6% of familial DCM cases, and these mutations often lead to a particularly severe form of the disease (Hey et al., 2019; Nishiyama et al., 2022). The target genes regulated by RBM20 are involved in various critical functions within cardiomyocytes, including sarcomere structure (e.g., TTN), calcium handling (e.g., RYR2 and CACNA1C) and mitochondrial function, as mentioned above (Beqqali et al., 2016; van den Hoogenhof et al., 2018; Kornienko et al., 2023). In DCM patients with variants in the RS-rich region of RBM20, the primary driver of their pathological phenotype appears to be the aberrant cellular localization of RBM20 (Kornienko et al., 2023). Interestingly, the precise genomic correction of RBM20 mutations has been shown to rescue the cardiac dysfunction in mice, offering potential benefits for DCM treatment (Conn et al., 2017).

Some other splicing factors involved in the development of DCM. Similar to RBM20, RBM24 is also involved in the regulation of alternative splicing of TTN. Knockout of RBM24 results in abnormal TTN splicing, which in turn leads to muscle structure disorders in cardiomyocytes and contributes to the subsequent development of DCM (Liu et al., 2019). The loss of hnRNPU expression results in aberrant splicing of the pre-mRNA encoding Junctin, an excitation-contraction coupling component, and ultimately leads to lethal DCM (Ye et al., 2015). In addition, Sam68-like mammalian protein 2 (SLM2) was reported to mediate intron inclusion of TTN mRNA in the myocardium of DCM patients (Boeckel et al., 2022). Furthermore, a study demonstrated thatAAv9-mediated overexpression of QKI5 prevented doxorubicin-induced apoptosis and expansion of cardiomyocytes in cardiomyopathy. QKI5 exerts its cardioprotective effects by regulating the expression of a specific set of circRNAs, including those derived from Titin, Fhod3, and Strn3 (Gupta et al., 2018). Despite the emerging evidence of the involvement of alternative splicing in DCM, the underlying molecular mechanisms and the specific contribution of RNA splicing to the pathogenesis of DCM still remain to be comprehensively explored.

### 4.2 Myocardial infarction

Myocardial infarction (MI) is the irreversible death of myocardial cells due to suddenly decrease in blood supply, which often leads to adverse remodeling of the left ventricular myocardium and ultimately to heart failure (Hasimbegovic et al., 2021). High-through sequencing has revealed changes in alternative splicing for a number of genes within the first 3 days after MI (Williams et al., 2018).

As the principal mediator of the hypoxic response after MI, hypoxia-inducible factor 1 (HIF-1) is considered to be involved in alternative splicing of metabolism and ion channel related genes in hypoxic or ischemic conditions. For instance, HIF-1 directly mediates a switch in pyruvate kinase isoforms from PKM1 to PKM2 after MI, and this isoform switch is likely to have significant consequences for ATP synthesis in infarcted cardiac muscle (Williams et al., 2018). Meanwhile, calcium/calmodulin dependent protein kinase II, gamma (CaMK2γ), a predominant isoform of the CaMK2, undergoes alternatively splicing and substantial downregulation after MI or in mouse heart carrying an oxygen-stable form of HIF-1α (HIF-PPN) (Williams et al., 2021). HIF1α could also activate SF3B1, a splicing factor mediating isoform switch of ketohexokinase from KHK-A to KHK-C in pathologic cardiac hypertrophy (Mirtschink et al., 2015). These evidences suggest that HIF-1α plays a significant role in the process of MI and pathologic cardiac hypertrophy by regulating RNA alternative splicing.

Insulin-like growth factor 1 (IGF-1) functions as a paracrine and autocrine growth factor and suppresses apoptosis in numerous cell types. Alternative splicing of the IGF-1 pre-mRNA gives rise to various isoforms carrying unique E-domains, whereas the minor isoform IGF-1Eb [also known as Mechano-Growth Factor (MGF)] is preferentially expressed in the heart following MI in mice (Mavrommatis et al., 2013). Both systemic and cardiac restricted delivery of the MGF E-domain peptide could restore the contractile function and prevent pathologic remodeling of the heart after MI (Pena et al., 2015). These data indicate a protective function of the E-domain region in response to myocardial ischemia. Nonetheless, the mechanism that governs IGF-1 splicing in the injured myocardium has yet to be elucidated.

Nicotinamide adenine dinucleotide (NAD^+^) has been demonstrated to boost cardiac angiogenesis and mitigate myocardial damage in diabetic mice following MI by increasing the ratio of pro-angiogenic VEGF_165_ to anti-angiogenic VEGF_165b_ in macrophages (Jiao et al., 2022). The factors mediating the role of NAD^+^ in VEGF splicing include SRSF1, which facilitates VEGF_165_ production, and SRSF6, which promotes VEGF_165b_ production. In addition, CircCDYL has emerged as a novel regulator of myocardial myogenesis following myocardial infarction, and may enhance cardiac function. CircCDYL was found to be significantly downregulated in both cardiac tissues and hypoxic cardiomyocytes. Overexpression of circCDYL promoted myocardial regeneration post-myocardial infarction by sponging miR-4793-5p, which in turn increased the expression levels of amyloid precursor protein (Li et al., 2020). Hence, the modulation of alternative splicing could be regarded as a potential approach for treating MI patients.

### 4.3 Heart failure

Heart failure (HF) represents the ultimate chronic stage of numerous cardiac diseases, characterized by an impairment in the efficient filling of the ventricles with blood or a reduced capacity to pump blood out of the ventricle (Ziaeian and Fonarow, 2016). The adult heart reverts to a fetal-like metabolic state and oxygen consumption in heart failure, which is associated with reactivation of fetal-specific RNA-binding proteins, and the accompanied re-expression of fetal-specific isoforms of genes such as SCN5A, TNNT2, and TTN (D’Antonio et al., 2022).

RBFOX1 is a prominent regulator of alternative RNA splicing during heart failure. The expression of RBFOX1 is significantly reduced in the failing heart, whereas cardiac deficiency of RBFOX1 results in heart failure in both mouse and zebrafish (Frese et al., 2015; Gao et al., 2016). Despite its global impact on cardiac mRNA splicing, the RBFOX1-mediated isoform switch (α1 versus α2) of MEF2 genes (MEF2A, MEF2C and MEF2D) appears to be a crucial regulatory circuit contributing to the development of heart failure (Gao et al., 2016). Significantly, cardiac re-expression of RBFOX1 substantially attenuated the manifestations in murine pressure overload models. Recently, splicing dysregulation in MEF2C towards another repressor variant, MEF2Cγ, had also been shown to contributes to the pathogenesis of heart failure by promoting cardiomyocyte dropout (Pereira et al., 2020). However, the factors responsible for controlling MEF2Cγ generation remain unknown.

Hu et al. identified a variant of the L-type voltage-gated calcium channel formed through alternative splicing, which includes of exons 21 and 22 (Cav1.2e21 + 22) (Hu et al., 2016). This isoform exhibits varying expression levels between neonatal and adult hearts, with a higher expression in neonates. Its presence is also elevated in a rat model of cardiac hypertrophy and heart failure. Importantly, this variant influences the presence of the wild-type calcium channels, leading to their degradation due to competitive interaction with the β subunit of the L-type voltage-gated calcium channels.

### 4.4 Atherosclerosis

Atherosclerosis is a primary contributor to several CVDs, such as MI, stroke and peripheral arterial disease. It is characterized by the formation of lipid-rich plaques within the inner layer of vascular wall in large and medium-sized arteries, originating from a chronic inflammatory response. Transcriptome-wide analysis uncovered a number of RBP-related alternative splicing events in the development of atherosclerosis (Wang et al., 2023).

Low and disturbed flow contribute to the progression of atherosclerosis by activating endothelial cells, leading to elevated expression of adhesion molecules, and facilitating the rolling, adhesion and extravasation of immune cells. Under conditions of low blood flow, murine arterial endothelium undergoes a series of alternative splicing events, including the inclusion of exons EIIIA and EIIIB in the extracellular matrix protein fibronectin (FN) (Murphy et al., 2018). The recruitment of platelets and macrophages to the arterial endothelium appears to be required for the flow-responsive splicing pattern in a large set of genes. Furthermore, endothelial deletion of RBFOX2, the only arterial endothelium-specific expressed family member, resulted in partial suppression of RNA splicing events and altered the intimal response to low flow (Murphy et al., 2018).

Tissue factor (TF), an integral-membrane glycoprotein involving in angiogenesis, is highly present in atherosclerotic plaques, and contributes to the plaque thrombogenicity upon rupture. In addition to the full-length transcript, an alternatively spliced isoform of TF (known as asTF) is also abundantly detected in the complicated human plaques but not in the un-complicated ones. This isoform is expressed primarily in macrophages and neovessels, promoting plaque angiogenesis through activation of HIF-1α and subsequent upregulation of soluble VEGF_165_ protein (Giannarelli et al., 2014). Meanwhile, asTF exhibits greater potency than its full-length isoform in inducing the expression of adhesion molecules in microvascular endothelial cells. This leads to an increased level of monocyte adhesion and transendothelial migration (Srinivasan et al., 2011).

Treg cells have been found in atherosclerotic plaques, and are considered to protect against clinical atherosclerosis. The forkhead box P3 (FOXP3), a key factor in the lineage specification of Treg cells, exists in various isoforms in humans such as the full-length FOXP3 (FOXP3fl) and FOXP3 lacking the region encoded by exon 2 (FOXP3Δ2). The activation of Treg cells results in significant upregulation of FOXP3Δ2, whereas low FOXP3Δ2 expression correlates with plaque instability (Joly et al., 2018). Although the underlying molecular mechanisms are unknown, FOXP3Δ2 seems to play a predominant role in Treg cells in suppressing the progression of atherosclerotic disease.

Accumulating evidence implicates non-coding RNAs in the development of CVDs. The antisense non-coding RNA in the INK4 locus (ANRIL) is a non-coding RNA closely related to the occurrence of atherosclerosis. The alternative splicing of ANRIL generates various linear and circular transcripts, which greatly involved in the expression of CAD-related genes (Gareev et al., 2022). While different linear ANRIL isoforms are attributed to opposing effects in both endothelial cells and vascular smooth cells during atherosclerosis, circular ANRIL isoforms are considered to protect atherosclerotic plaques and reduce atherosclerosis risk (Cho et al., 2020; Gareev et al., 2022; Mayner et al., 2023).

## 5 Conclusion and perspectives

The concept of alternative splicing, although known for many decades, has gained significant recognition only in recent years, owing to the emergence of high-throughput techniques and advanced analytical methods. We are now beginning to comprehend the extensive influences of alternative splicing on the pathophysiology of various conditions. A growing number of CVDs are associated with alterations in RNA splicing patterns, which are under the coordinated regulation of multiple RBPs, either cooperatively or antagonistically. This makes them promising targets for therapeutic interventions.

Apart from mouse models and primary cell cultures, human cardiovascular cells and cardiovascular organoids, derived from the induced pluripotent stem cells (iPSCs) and embryonic stem cells (ESCs), are emerging as ideal models for investigating the mechanisms underlying alternative splicing in cardiac development and the pathophysiology of CVDs. Additionally, CRISPR/Cas9-based gene editing has become a powerful tool for studying and potentially correcting genetic abnormalities associated with genetic cardiomyopathies. With the advancements in single-cell sequencing technology, we can look forward to the development of highly effective sequencing strategies for identification of splicing-related diseases, and providing novel targets for personalized diagnosis and treatment of CVDs.
